# Cell-Intrinsic Role for NF-kappa B-Inducing Kinase in Peripheral Maintenance but Not Thymic Development of Foxp3^+^ Regulatory T Cells in Mice

**DOI:** 10.1371/journal.pone.0076216

**Published:** 2013-09-20

**Authors:** Susan E. Murray

**Affiliations:** Department of Molecular Microbiology and Immunology, Oregon Health & Science University, Portland, Oregon, United States of America; Escola Paulista de Medicina - UNIFESP, Brazil

## Abstract

NF-κB inducing kinase (NIK, MAP3K14) is a key signaling molecule in non-canonical NF-κB activation, and NIK deficient mice have been instrumental in deciphering the immunologic role of this pathway. Global ablation of NIK prevents lymph node development, impairs thymic stromal development, and drastically reduces B cells. Despite altered thymic selection, T cell numbers are near normal in NIK deficient mice. The exception is CD4^+^ regulatory T cells (Tregs), which are reduced in the thymus and periphery. Defects in thymic stroma are known to contribute to impaired Treg generation, but whether NIK also plays a cell intrinsic role in Tregs is unknown. Here, we compared intact mice with single and mixed BM chimeric mice to assess the intrinsic role of NIK in Treg generation and maintenance. We found that while NIK expression in stromal cells suffices for normal thymic Treg development, NIK is required cell-intrinsically to maintain peripheral Tregs. In addition, we unexpectedly discovered a cell-intrinsic role for NIK in memory phenotype conventional T cells that is masked in intact mice, but revealed in BM chimeras. These results demonstrate a novel role for NIK in peripheral regulatory and memory phenotype T cell homeostasis.

## Introduction

NF-κB is an evolutionarily conserved intracellular signaling pathway that acts as a critical immune sensor. Canonical NF-κB mediates cellular responses to myriad danger and inflammatory signals including pattern recognition receptors, antigen receptors, and cytokine and chemokine receptors. This pathway is activated rapidly—within minutes of receptor ligation—by virtue of rapid phosphorylation and degradation of inhibitory IκB proteins that retain the transcriptionally active NF-κB subunits in the cytosol. In contrast, non-canonical NF-κB is activated more slowly, as it requires new protein synthesis, and it is not dependent on IκB degradation [1]. Instead, it relies on accumulation of NF-κB inducing kinase (NIK) and subsequent phosphorylation of IKKα, which induces partial proteasomal degradation of the NF-κB2 subunit. This releases active dimers of p52:RelB from the cytosol to the nucleus to allow gene transcription. In addition, unlike the canonical pathway, activation of non-canonical NF-κB is restricted to a subset of TNF receptor family members (TNFR). In particular, this pathway is important for lymphoid organogenesis downstream of LTβR and for B cell survival downstream of BAFFR [2-4]. In addition, NIK and NF-κB2 expression by stromal cells are necessary for development of normal thymic epithelium [5-7], and their absence in thymic stroma impairs negative selection of autoreactive T cells and generation of regulatory T cells [8,9]. More recently, NIK has been shown to play T cell-intrinsic roles in mouse models of autoimmunity [10,11], and we and others have shown that NIK is critical downstream of the costimulatory TNFR, OX40, for Th1 and Th9 effector function [12,13]. In addition, we recently found that CD4^+^ regulatory T cells overexpressing NIK have impaired suppressive function [12].

CD4^+^Foxp3^+^ regulatory T cells (Tregs) are essential negative regulators of the adaptive immune response. Their absence in mice and humans causes lethal multiorgan autoimmunity [14-17]. Treg proportions are decreased in NIK-deficient mice, but this has been attributed to i) altered thymic stroma as described above [9], and ii) altered peripheral antigen presenting cell (APC) function [18]. Recently, the canonical NF-κB subunit, c-Rel, was discovered to play an essential cell-intrinsic role in thymic Treg development [19-21], but no one has investigated whether non-canonical NF-κB plays a cell-intrinsic role in thymic Treg development or peripheral Treg homeostasis.

Here, we challenge the conclusion that Treg alterations caused by NIK-deficiency are all secondary to effects on stromal cells and APC. We found that while NIK expression in stromal cells is sufficient to generate normal proportions and numbers of thymic Tregs, NIK plays an essential cell-intrinsic role in peripheral Treg maintenance. In addition, we found significantly decreased proportions of memory phenotype conventional CD4^+^ T cells in the absence of NIK, an effect which is also cell intrinsic. These data identify a previously unappreciated cell-intrinsic role for NIK in peripheral Treg and memory phenotype T cell homeostasis.

## Materials and Methods

### Ethics statement

All procedures were approved by the Oregon Health & Science University Institutional Animal Care and Use Committee under protocol number A378 to David C. Parker.

### Mice

NIK KO mice were from R. Schreiber (Washington University School of Medicine) [3]. B6.CD45.1 mice were from The Jackson Laboratory (B6.CD45.1xB6.CD45.2). F1 mice were bred in house. Foxp3-RFP mice were from The Jackson Laboratory and were bred with NIK KO mice to homozygosity.

### Bone Marrow (BM) chimeras

BM was harvested from femurs and tibias of 2-3 month-old NIK KO, WT littermate, (B6.CD45.1xB6.CD45.2) F1, Foxp3-RFP NIK KO, or Foxp3-RFP WT littermate mice. 1x10^6^ BM cells were injected intravenously into lethally irradiated B6.CD45.1 recipients (6 Gy whole body γ irradiation x 2). Recipients were treated with ciprofloxacin in the drinking water for 4 weeks post-reconstitution. For mixed BM chimeras, 0.5x10^6^ each of i) NIK KO or WT littermate (CD45.2) and ii) (B6.CD45.1xB6.CD45.2) F1 cells were injected. T cells were analyzed 8-16 weeks later.

### Antibodies and flow cytometry

Fluorochrome-conjugated antibodies were from BioLegend and eBioscience: CD4 (RM4-5), CD8 (53-6.7), CD25 (PC61.5), CD44 (IM7), CD45.1 (A20), CD45.2 (104), CD62L (MEL-14), CD152/CTLA4 (UC10-4B9), Foxp3 (FJK-16s). Nucleated thymus, spleen, and lymph node cell suspensions were stained for surface markers and intracellular proteins as previously described [12]. Cells were analyzed on an LSR II flow cytometer (BD Biosciences) and analyzed using FlowJo (Tree Star). RFP^+^ cells were sorted on an Influx (BD Biosciences). The intracellular proteins, Foxp3 and CTLA-4, were stained after fixation and permeabilization following manufacturer’s instructions (Biolegend Foxp3 staining kit).

### In vitro iTreg differentiation

CD25-depleted CD4^+^ T cells were magnetically purified from spleens of NIK KO or WT BM chimeras using EasySep mouse CD4^+^ T cell enrichment kit (Stem Cell Technologies) with the addition of anti-CD25-biotin to the biotinylated antibody cocktail. Cells were stimulated with immobilized anti-CD3 and anti-CD28 (5µg/ml each) and cultured with 2-8ng/ml TGFβ, 100U/ml IL-2 and with or without 10nM all-trans retinoic acid, blocking anti-IFNγ antibody (MG1.2), and blocking anti-IL-4 antibody (11B11).

### Treg suppression assay

CD4^+^RFP^+^ cells (Tregs) were sorted from spleens of Foxp3-RFP NIK KO and Foxp3-RFP WT littermate BM chimeric mice. CD25-depleted CD4^+^ T cells (Tconv) were magnetically purified from spleens of WT mice using EasySep mouse CD4^+^ T cell enrichment kit (Stem Cell Technologies) with the addition of anti-CD25-biotin to the biotinylated antibody cocktail. Tregs were labeled with CFSE as previously described [12], and Tconv were labeled with CellTrace Violet proliferation dye per manufacturer’s instructions (Invitrogen). CD45.1^+^ APC were prepared by red blood cell lysis and 10 Gy γ irradiation of single-cell spleen suspensions. 5 × 10^4^ Tconv were cultured with 1 × 10^5^ APC, varying numbers of Tregs, and 0.5 µg/ml soluble anti-CD3 for 3 days. Cell division of Tregs and Tconv was assessed by flow cytometry.

### Statistics

WT and NIK KO group means were compared by unpaired Student’s t-test. *, p<0.05. ns, not significant.

## Results

Tregs (assessed in previous reports as CD4^+^CD25^+^) are decreased in the thymus and spleen of NIK KO and NIK^aly^ mice, which harbor a loss of function mutation in NIK [9,18,22]. Although NIK deficiency only in the thymic stroma suffices to produce CD4^+^ T cell-mediated autoimmunity and altered Treg generation [9], it is not known whether NIK also plays a cell-intrinsic role in thymic Treg generation or peripheral Treg maintenance. We compared intact NIK KO mice, single NIK KO BM chimeras, and mixed (WT+NIK KO) BM chimeras to answer this question.

### Foxp3^+^ Tregs and CD25^+^Foxp3^-^ precursors are decreased in NIK KO thymus

**Figure 1:**
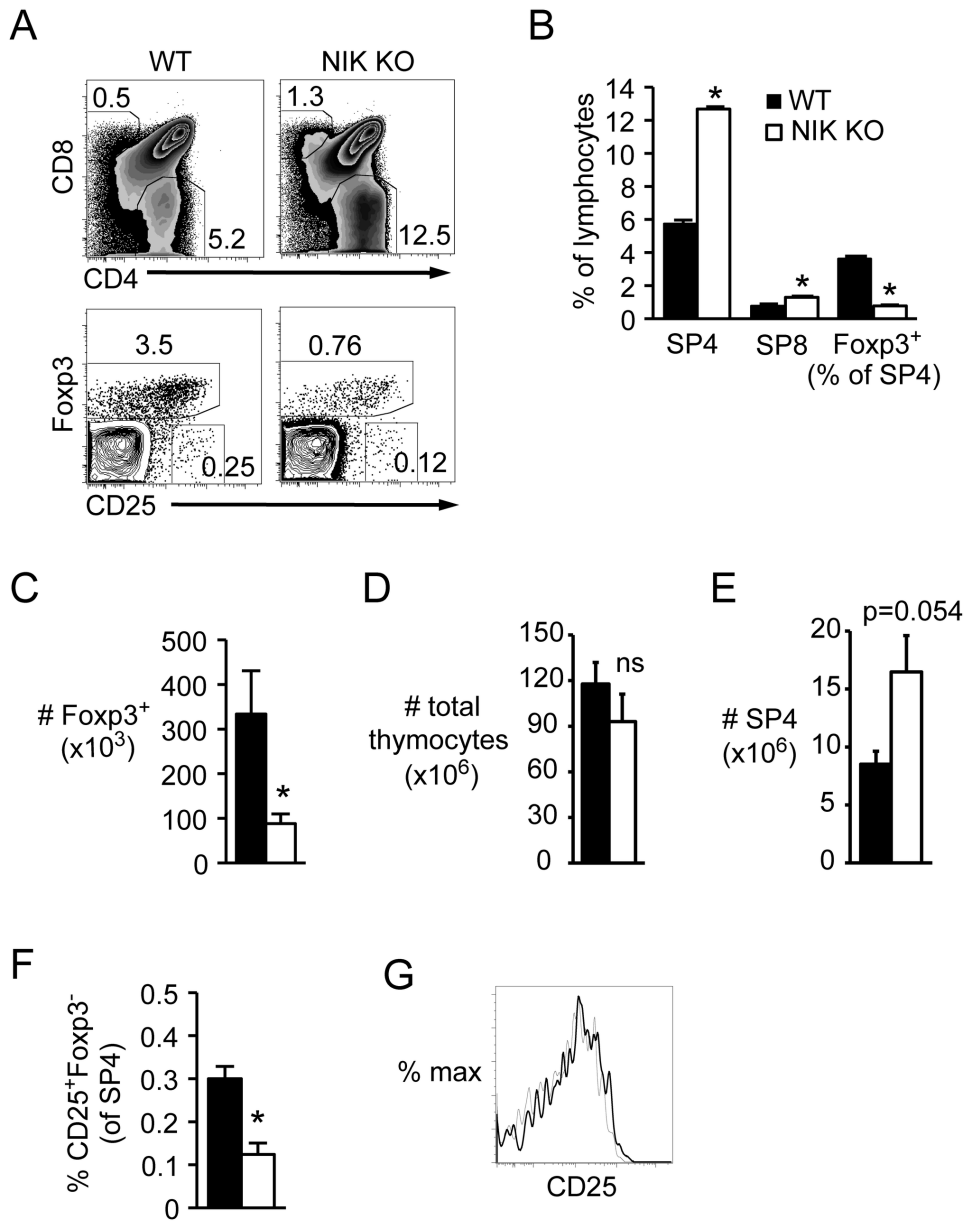
Decreased Foxp3^+^ Tregs and CD25^+^Foxp3^-^ Treg precursors in NIK KO thymus. Single-cell suspensions of nucleated thymocytes from intact NIK KO or WT mice were stained with the indicated fluorescently conjugated antibodies and analyzed by flow cytometry. A, Top plots are gated on lymphocytes, bottom plots are gated on SP4. Numbers indicate percent of gated cells. B, Quantitation of cell percentages among lymphocytes or SP4. C-E, Quantitation of absolute number of SP4 Foxp3^+^ thymocytes, total thymocytes, and SP4 thymocytes per mouse, respectively. F, Quantitation of Treg precursors as a percent of SP4. G, Overlay of CD25 expression gated on SP4 Foxp3^+^ cells; bold line, NIK KO; narrow line, WT. Data in bar graphs represent mean ± SEM of n=4 mice per group in one representative experiment of two.

Using Foxp3 as a specific Treg marker, we found that the proportion of Tregs among CD4 single positive cells (SP4) was decreased in NIK KO thymus (Figure 1A and B). Although this was partially offset by the increased number and proportion of SP4 in NIK KO thymus (Figure 1A, B, and E), the total number of Foxp3^+^ SP4 was still significantly decreased (Figure 1C) while total thymic cellularity was normal (Figure 1D). The proportion of CD25^+^Foxp3^-^ Treg precursors was also significantly decreased in NIK KO thymus (Figure 1A and F), although CD25 expression was equivalent between NIK KO and WT Foxp3^+^ cells (Figure 1A and G).

### WT recipients of NIK KO BM have normal thymic Tregs

**Figure 2:**
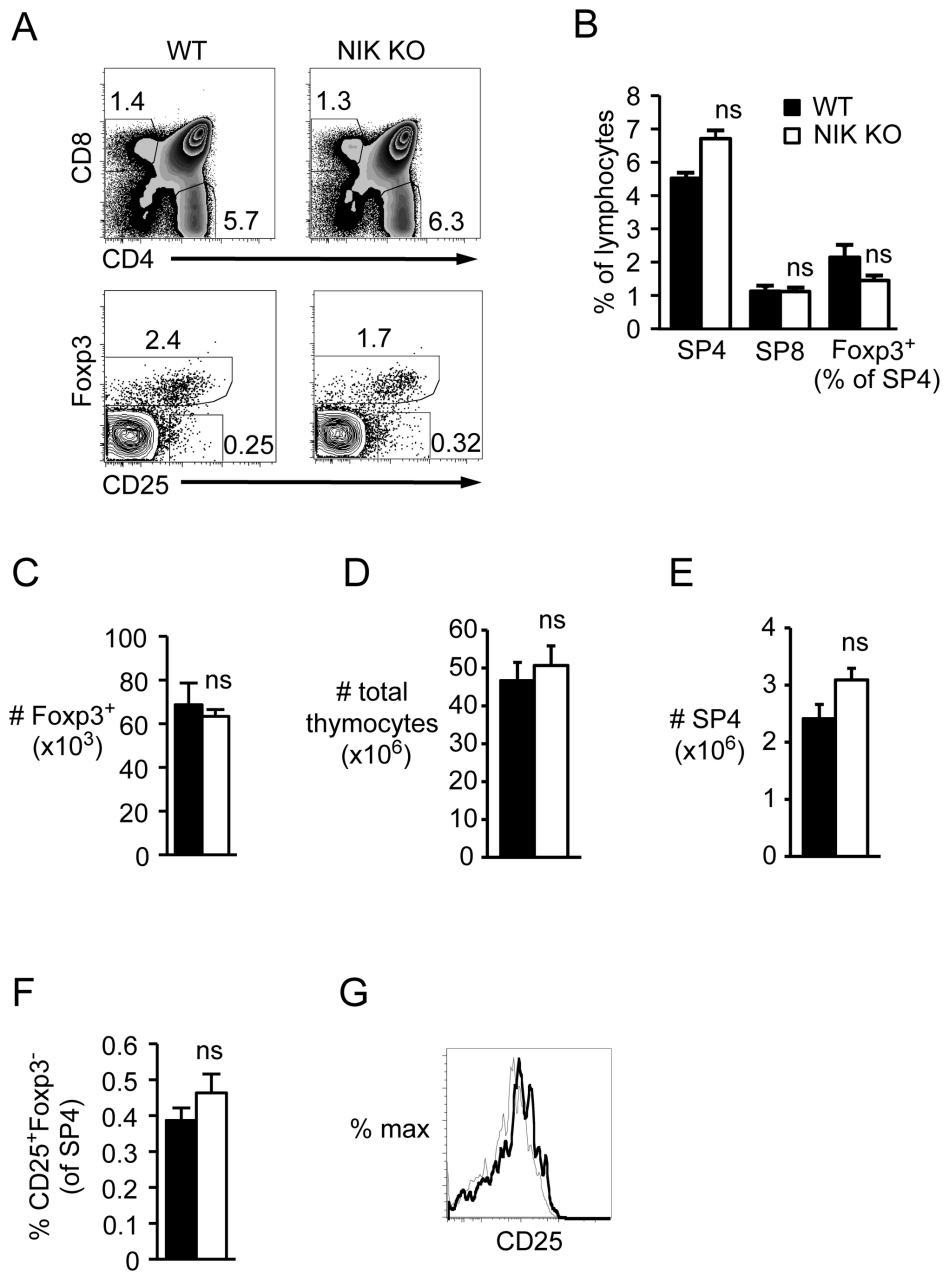
Normal Foxp3^+^ Tregs and CD25^+^Foxp3^-^ Treg precursors in thymus of WT recipients of NIK KO BM. Single-cell suspensions of nucleated thymocytes from NIK KO or WT BM chimeric mice were stained with the indicated fluorescently conjugated antibodies and analyzed by flow cytometry. A, Top plots are gated on donor CD45.1^-^ lymphocytes (BM derived, NIK KO or WT littermate); bottom plots are gated on CD45.1^-^ SP4. Numbers indicate percent of gated cells. B, Quantitation of cell percentages among CD45.1^-^ lymphocytes or SP4. C-E, Quantitation of absolute number of SP4 Foxp3^+^ CD45.1^-^ thymocytes, total CD45.1^-^ thymocytes, and SP4 CD45.1^-^ thymocytes per mouse, respectively. F, Quantitation of Treg precursors as a percent of CD45.1^-^ SP4. G, Overlay of CD25 expression gated on CD45.1^-^ SP4 Foxp3^+^; bold line, NIK KO; narrow line, WT. Data in bar graphs represent mean ± SEM of n=3-4 mice per group in one representative experiment of two.

In contrast, thymic SP4 (Figure 2A, B, and E) and thymic Treg percentages and numbers (Figure 2A-C) were normalized in WT recipients of NIK KO BM, as were CD25^+^Foxp3^-^ Treg precursors (Figure 2A and F). Similar to intact mice, NIK KO Tregs expressed normal levels of CD25 (Figure 2A and G). These data clearly show that thymic Treg perturbations depend on NIK expression in non-hematopoietic cells, as previously suggested [9].

### NIK KO peripheral Treg paucity is cell-intrinsic

**Figure 3:**
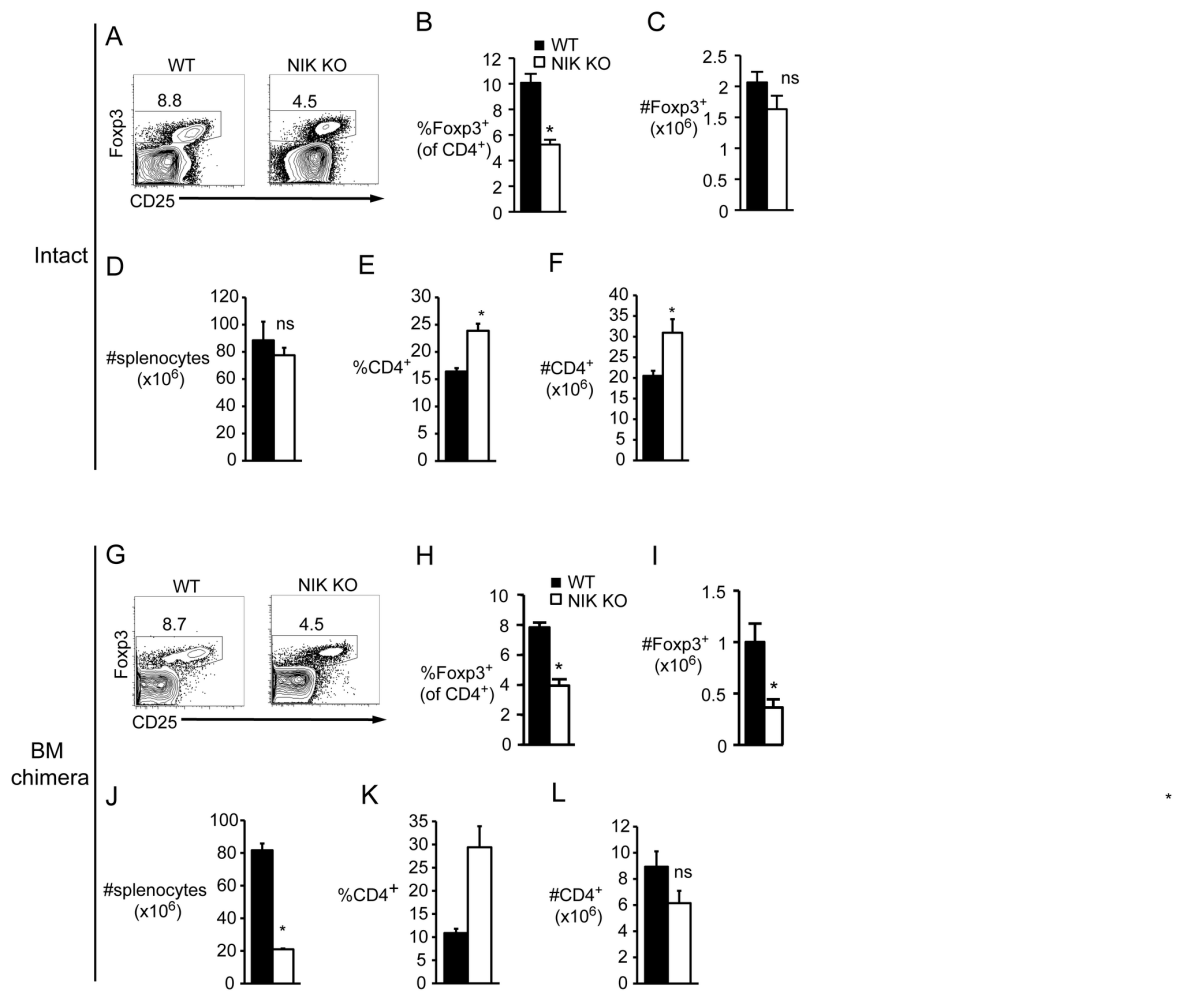
Decreased peripheral Foxp3^+^ Tregs in both intact NIK KO and NIK KO single BM chimeric mice. Single cell suspensions of nucleated splenocytes from intact (A-F) or BM chimeric (G-L) mice were stained with the indicated fluorescently labeled antibodies. A, Plots are gated on CD4^+^; numbers indicate percent of gated cells. B and E, Quantitation of percent of Foxp3^+^ and CD4^+^ splenocytes, respectively. C, D, and F, Quantitation of absolute number of Foxp3^+^, total, and CD4^+^ splenocytes. G, Plots are gated on CD4^+^CD45.1^-^ cells (BM-derived, NIK KO or WT littermate). Numbers indicate percent of gated cells. H and K, Quantitation of percent of Foxp3 ^+^ CD45.1^-^ and CD4^+^CD45.1^-^ cells, respectively. I, J, and L, Quantitation of absolute number of Foxp3 ^+^ CD45.1^-^, total CD45.1^-^, and CD4^+^CD45.1^-^ splenocytes, respectively. Data in bar graphs represent mean ± SEM of n=3-4 mice per group in one representative experiment of two (B-F) or three (G-L).

A very different picture emerged in the periphery. As in the thymus, Treg percentages were decreased in NIK KO spleen (Figure 3A and B), but Treg numbers were normal (Figure 3C) due to an overall increase in CD4 T cell percent and number (Figure 3E and F) that was sufficient to overcome the expected loss in splenic cellularity due to poor B cell survival (Figure 3D). However, unlike in the thymus, peripheral Treg deficits were not reversed in WT recipients of NIK KO BM. The percent of Foxp3^+^ cells was decreased to the same degree as in intact NIK KO mice (Figure 3G and H), and since the number of total CD4 T cells was unchanged (Figure 3L), the number of Tregs was also significantly decreased (Figure 3I). Note that the splenic cellularity and CD4 T cell proportion are altered secondary to the well-known requirement of NIK for B cell survival (Figure 3J and K). One possible explanation for decreased peripheral NIK KO Tregs could be inefficient differentiation from conventional T cells. We tested whether NIK KO T cells differentiate normally in vitro and found that under a variety of Treg-inducing conditions, the proportion and number of NIK KO conventional T cells that upregulated Foxp3 was indistinguishable from WT (Figure S1).

### Altered Treg proportions and numbers in mixed BM chimeras

**Figure 4:**
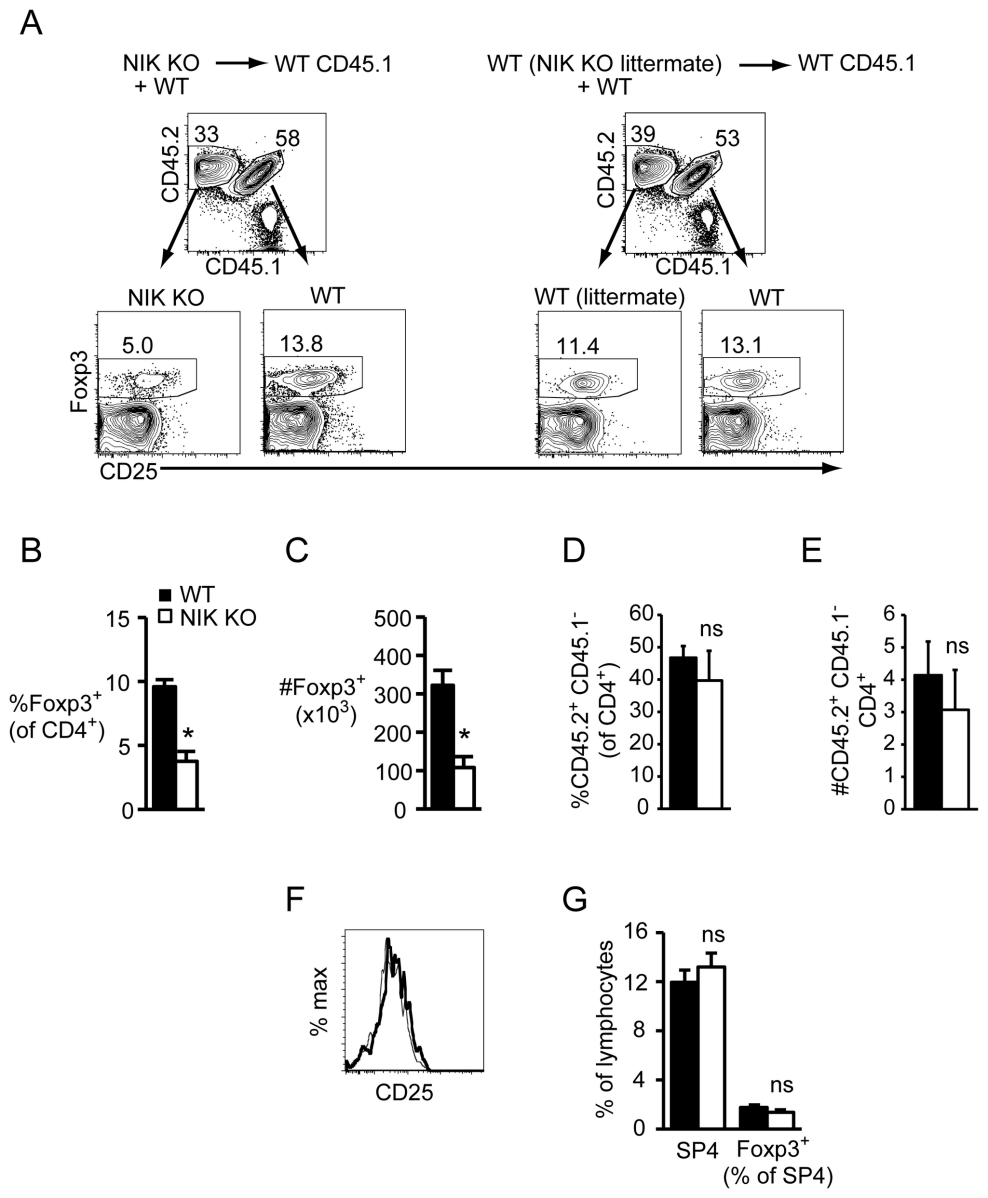
Decreased peripheral Foxp3^+^ Tregs in NIK KO BM chimeric mice is cell-intrinsic. Single cell suspensions of nucleated splenocytes (A-F) or thymocytes (G) from NIK KO (CD45.2) + WT (CD45.1xCD45.2) F1 or WT littermate (CD45.2) + WT (CD45.1xCD45.2) F1 mixed BM chimeric mice were stained with the indicated fluorescently labeled antibodies. A, Gating scheme for identifying cells in mixed BM chimeras. CD45.1 ^+^ CD45.2^-^ cells (ungated) are radioresistant host cells. Numbers indicate percent of gated cells. B and C, Quantitation of percent and absolute number of donor CD45.2 ^+^ CD45.1^-^Foxp3^+^ cells. D and E, Quantitation of percent and absolute number of donor CD45.2 ^+^ CD45.1^-^CD4^+^ cells. These data compare NIK KO cells with WT littermate control cells. Because WT (CD45.1xCD45.2) F1 cells appear to have a slight, but consistent advantage over CD45.2 ^+^ CD45.1^-^ WT littermates in terms of both overall proportion and proportion of Foxp3^+^ cells, comparing NIK KO T cells to WT littermate T cells is the most conservative and appropriate comparison. When we compare NIK KO T cells to WT (CD45.1xCD45.2) F1 T cells, the differences are even greater. F, CD25 expression on NIK KO (bold line) versus WT (dashed line) Foxp3^+^ splenocytes. G, Quantitation of percent of NIK KO and WT SP4 and Foxp3^+^ thymocytes in mixed BM chimeras. Data in bar graphs represent mean ± SEM of n=3-4 mice per group in one representative experiment of two.

Poorly costimulatory dendritic cells were previously suggested to contribute to the Treg deficit in NIK KO mice [18]. If this were the case, we would expect the NIK KO Treg defect to be abrogated in mixed NIK KO + WT BM chimeras because WT dendritic cells are present. However, similar to intact NIK KO and single BM chimeric mice, NIK KO Tregs were decreased over 2-fold in mixed BM chimeras (Figure 4A-C), despite the fact that proportions and numbers of total NIK KO CD4 T cells were normal (Figure 4D and E). Again, the decrease in NIK KO Tregs was not associated with decreased CD25 expression (Figure 4F). We observed a similar magnitude decrease in peripheral and mesenteric lymph node Tregs in both single and mixed BM chimeras (Figure S2). Since mixed BM chimeras have equal proportions of WT and NIK KO thymic Tregs (Figure 4G), we conclude that NIK is required cell-intrinsically to maintain peripheral Tregs.

### NIK KO Treg phenotype and function

**Figure 5:**
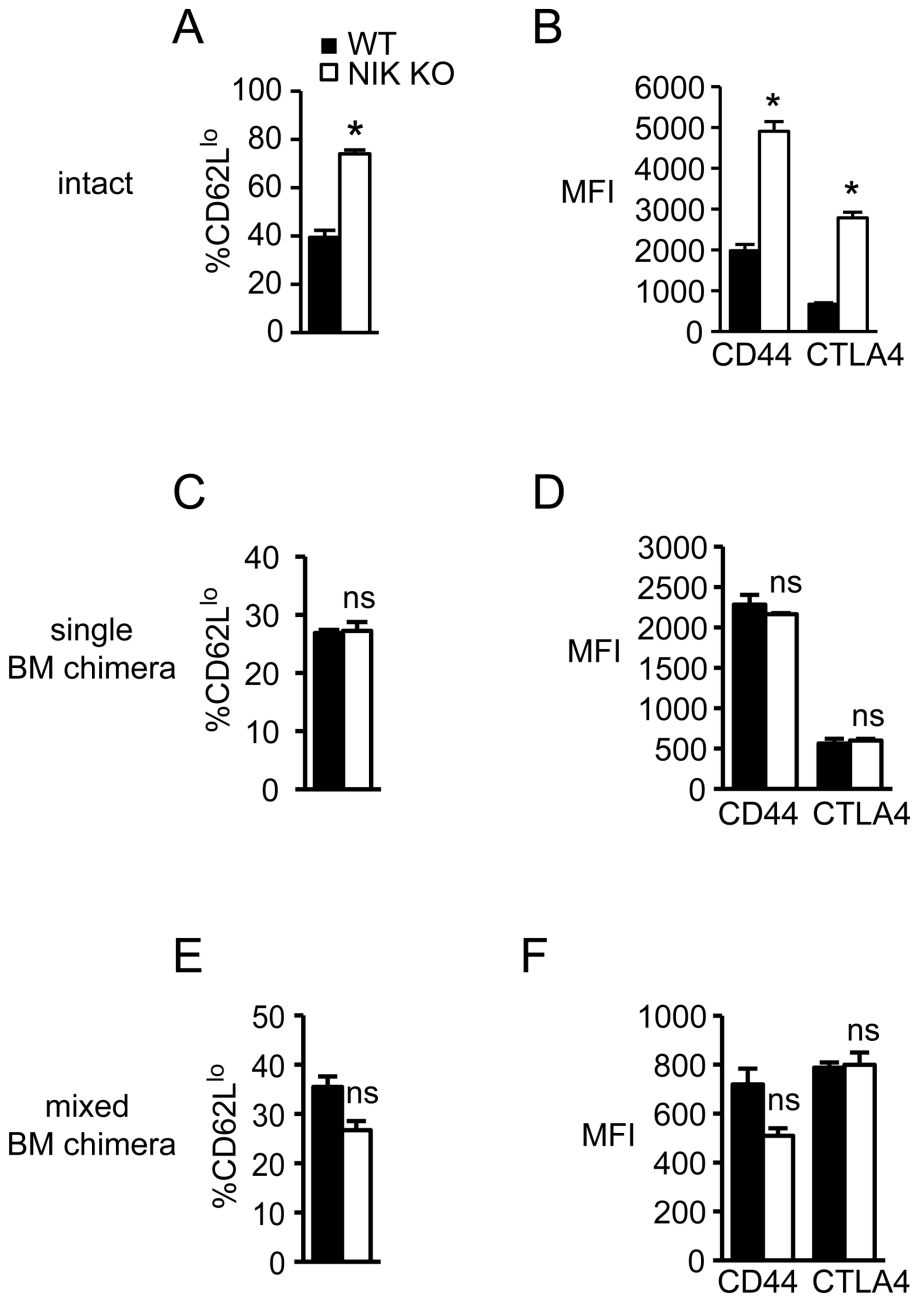
Altered phenotype of NIK KO Tregs depends on NIK KO stromal cells. Single cell suspensions of nucleated splenocytes from intact (A and B), single BM chimeric (C and D), or mixed BM chimeric (E and F) mice were stained with the indicated fluorescently labeled antibodies. A, C, and E, Quantitation of percent CD62L^lo^ among Foxp3^+^ NIK KO or WT cells. B, D, and F, Quantitation of mean fluorescence intensity of CD44 and CTLA4 on Foxp3^+^ NIK KO or WT cells. Data represent mean ± SEM of n=3-4 mice per group in one representative experiment of two (A, B, E, F) or three (C, D).

A previous report described an increased CD62L^lo^ population among NIK KO CD25^+^ cells, and the presence of these activated Tregs (which also expressed increased CD69, CD45RB, and CD44) was suggested to contribute to autoimmunity [22]. Using Foxp3 to identify Tregs, instead of CD25 as was used in the previous report [22], we also observed an increased proportion of CD62L^lo^ cells within the splenic NIK KO Treg population (Figure 5A). In addition, we found substantially increased expression of CD44 and CTLA4 on NIK KO Tregs (Figure 5B), showing that despite their paucity, NIK KO Tregs display an activated phenotype. However, this activated phenotype was neither Treg intrinsic nor related to effects in other hematopoietic cells, because it disappeared in single and mixed BM chimeras (Figure 5C-F). Thus, these changes are likely secondary to autoimmune inflammation driven by altered T cell selection when stromal cells lack NIK. Consistent with their normal phenotype, NIK KO Tregs that develop in a NIK-sufficient host suppress conventional T cell proliferation equally to WT Tregs on a per cell basis (Figure S3A and B). In addition, NIK KO Tregs themselves proliferate equally to WT Tregs in vitro (Figure S3C and D).

### Cell-intrinsic role for NIK in memory phenotype T cells

**Figure 6:**
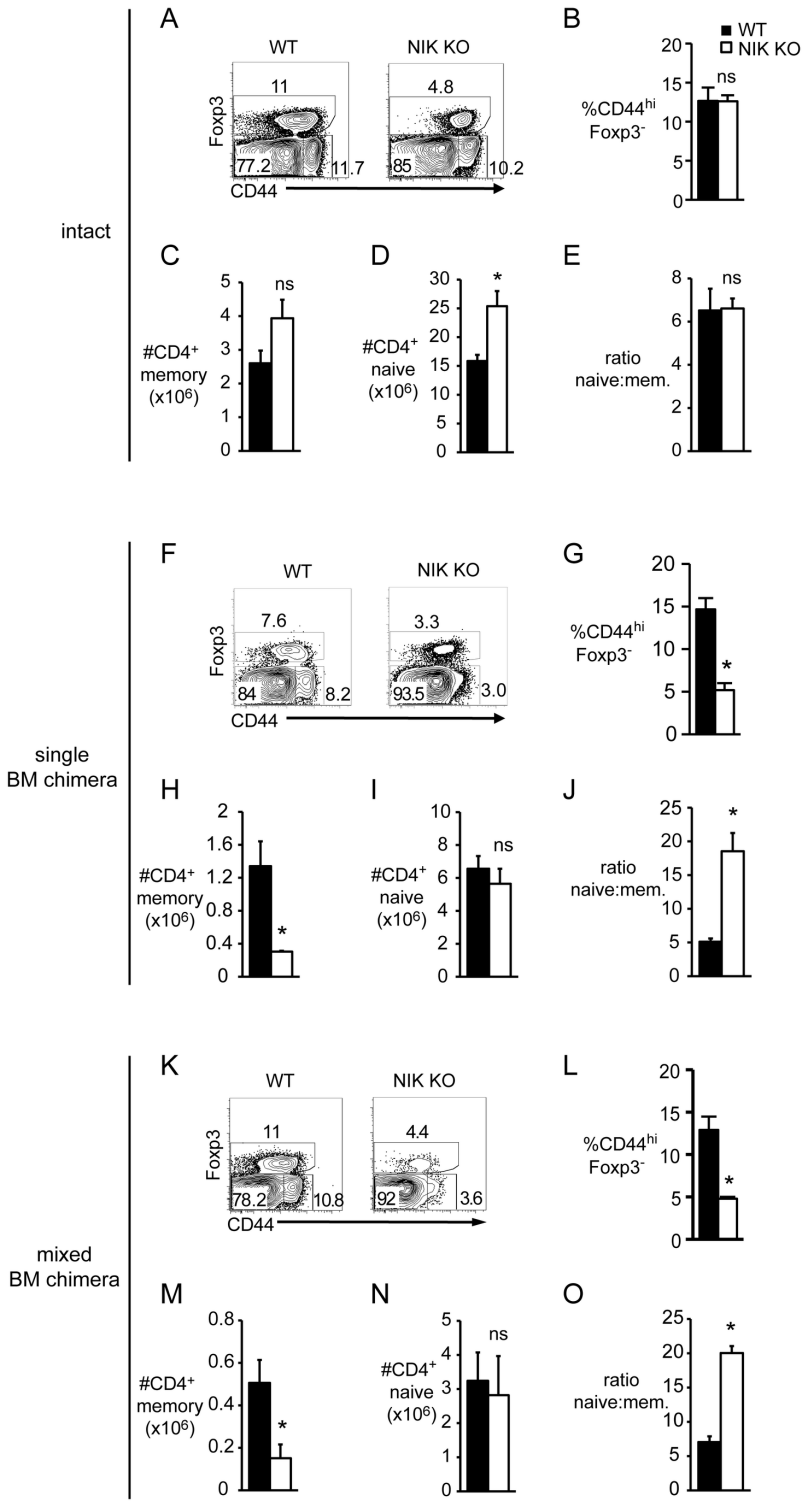
NIK is required cell-intrinsically for normal proportions of memory phenotype conventional T cells. Single cell suspensions of nucleated splenocytes from intact (A-E), single BM chimeric (F-J), or mixed BM chimeric (K-O) mice were stained with the indicated fluorescently labeled antibodies. Dot plots are gated on CD4^+^ (A), CD4^+^CD45.1^-^ (F), or CD4^+^CD45.1^-^CD45.2^+^ (K) cells. Numbers on plots indicate percent of gated cells. B, G, and L, Quantitation of percent Foxp3^-^CD44^hi^ within the gated populations described in A, F, and K, respectively. C, H, and M, Quantitation of absolute number of CD4^+^ memory T cells of the indicated genotype per spleen. D, I, and N, Quantitation of absolute number of CD4^+^ naive T cells of the indicated genotype per spleen. E, J, and O, Quantitation of naïve: memory CD4^+^ T cell ratio of the indicated genotype. Data in bar graphs represent mean ± SEM of n=3-4 mice per group in one representative experiment of two (A-E and K-O) or three (F-J).

In an interesting parallel observation, we found that NIK KO conventional T cells (CD4^+^Foxp3^-^) from intact mice have normal numbers and proportions of CD44^lo^ and CD44^hi^ populations (Figure 6A-E), but in both single and mixed BM chimeras the number and proportion of these memory phenotype cells is significantly decreased (Figure 6F-H and K-M). In contrast, naïve CD4 T cell numbers are normal (Figure 6I and N), leading to a skewed naïve: memory ratio (Figure 6J and O). Thus, NIK seems to be intrinsically important for generation or maintenance of memory phenotype T cells, but this requirement is masked in intact NIK KO mice, likely by virtue of expansion of this population due to autoimmunity.

## Discussion

As a key kinase in the non-canonical NF-κB signaling pathway, NIK is essential for lymphoid organ development and B cell survival. Because of severe defects in these compartments in NIK KO mice, and because NIK KO mice have grossly normal numbers of most peripheral T cell compartments, appreciation that NIK plays an intrinsic role in T cells has been slow to evolve. Only recently has NIK been shown to signal downstream of TNFRs on CD4 T cells during immune responses [11-13]. In naïve NIK KO mice, the T cell abnormalities—autoreactive conventional T cells and a paucity of CD25^+^ Tregs—were attributed to the known effects of NIK on thymic epithelium or APC [8,9,18]. Here, we found that NIK is indeed dispensable in Tregs for thymic development, and is required only in non-hematopoietic cells. However, despite normal thymic development, peripheral Tregs were significantly decreased when only hematopoietic cells lack NIK, showing that this effect cannot be attributed to altered thymic epithelium. In addition, we saw decreased proportions of NIK KO Tregs in mixed BM chimeras, where NIK KO and WT T cells enjoy the same ratio of WT:NIK KO APC and other accessory cells. Similarly, we found a cell-intrinsic requirement for generation or maintenance of conventional T cells with a memory phenotype. Separate studies from our laboratory have demonstrated a cell-intrinsic requirement for NIK in the generation or maintenance of antigen-specific CD4^+^ and CD8^+^ memory T cells [23].

Despite decreased numbers of NIK KO Tregs, NIK deficiency does not seem to intrinsically impair Treg function on a per cell basis as assessed by their ability to suppress conventional T cell proliferation (Figure S3). Likewise, Treg proliferation in the presence of activated conventional T cells is also normal in NIK KO T cells (Figure S3), as is Treg differentiation from conventional T cells (Figure S1). These findings, combined with normal thymic Treg output, suggest that NIK plays a role in peripheral Treg survival or homeostatic turnover, rather than differentiation. It is as yet unclear which receptor(s) might mediate this effect. Tregs depend on IL-2 for survival, but CD25 expression was not different between NIK KO and WT Tregs (Figure 4F), and IL-2R has not been described to activate NF-κB. CD28 provides a signal required for both Treg development [24] and peripheral survival [25]. Signaling through CD28 was recently shown to depend, in part, on NIK-mediated phosphorylation of c-Rel [26], a canonical NF-κB subunit that is also required for Treg development [19-21]. If deficient signaling through CD28 underlies the decrease in NIK KO Tregs, it is curious that we see a cell-intrinsic effect in the periphery, but not in the thymus, given that CD28 is also crucial for Treg development [24]. It may be that by inhibiting only one of many CD28-activated pathways, sufficient signaling is preserved to enable efficient thymic Treg development, but not peripheral maintenance.

Another non-mutually exclusive possibility is impaired signaling through one or more TNFR family members, several of which are known to activate non-canonical NF-κB. Tregs constitutively express OX40, which we previously showed signals through NIK in Tregs differentiated in vitro [12]. OX40 KO mice have fewer peripheral Tregs early in life [27], and although numbers in spleen and lymph nodes normalize in adulthood, those in the gut do not [28]. Likewise, peripheral Tregs are decreased in GITR KO mice [29]—another TNFR expressed constitutively by Tregs. Moreover, increased Tregs in GITRL transgenic mice were attributed to increased homeostatic proliferation [30]. Although NIK signaling has not been investigated downstream of GITR in Tregs, in transfected cell lines GITR induces non-canonical NF-κB activation [31]. Curiously, NIK KO Tregs respond hyperproliferatively to in vitro GITR ligation [22], but this was shown to result from the increased activation state of NIK KO Tregs, which we now conclude is secondary to autoimmunity resulting from altered thymic stroma. Future studies should evaluate the role of NIK in OX40 and GITR-mediated Treg homeostasis in BM chimeras to exclude effects of altered thymic stroma.

There is great interest in therapeutic manipulation of Tregs to ameliorate autoimmunity or maintain transplant tolerance on the one hand, or to augment cancer immunotherapy on the other. Likewise, non-canonical NF-κB has been proposed as an attractive target for immune modulation [32-34]. Here, we conclusively demonstrate that NIK plays an essential cell-intrinsic role in peripheral Treg maintenance that is unrelated to its role in thymic stroma. This suggests that drugs designed to inhibit non-canonical NF-κB in patients may reduce Treg numbers, an outcome which could conflict with the desired immunosuppressive effects.

## Supporting Information

Figure S1 **Normal in vitro Treg differentiation from NIK KO conventional T cells.** CD25-depleted conventional CD4^+^ T cells isolated from BM chimeric mice were differentiated in vitro for 3 days under the indicated conditions. In all conditions cells were stimulated with immobilized anti-CD3 + anti-CD28 and supplemented with IL-2. Cultures were analyzed for percent (A) and number (B) of Foxp3 ^+^ CD4^+^ T cells.
(TIF)

Figure S2 **Decreased proportion of NIK KO Tregs in mesenteric and peripheral lymph nodes of single and mixed BM chimeric mice.** Single cell suspensions of pooled inguinal, axillary, and brachial LN (pLN) or mesenteric LN (mLN) from single or mixed BM chimeric mice were stained with fluorescently labeled antibodies to CD4, Foxp3, CD45.1, and CD45.2. A, Quantitation of the proportion of CD4^+^CD45.1^-^ cells that are Foxp3^+^. B, Quantitation of the proportion of CD4^+^CD45.1^-^CD45.2^+^ cells that are Foxp3^+^. As in Figure 4, these data compare NIK KO cells with WT littermate control cells.
(TIF)

Figure S3 **Normal suppressive capacity and proliferation by NIK KO Tregs.** Foxp3-RFP ^+^ CD4^+^ cells were FACS-sorted from spleens of WT BM chimera recipients that had received Foxp3-RFP NIK KO or Foxp3-RFP WT BM 8 weeks earlier. Sorted Tregs were labeled with CFSE and plated at varying ratios with CD25-depleted CD4^+^ Tconv labeled with CellTrace Violet proliferation dye. Cells were stimulated for 3 days with irradiated CD45.1^+^ splenocytes as APC and soluble anti-CD3. Tconv and Treg cell division was assessed by flow cytometry. A and B, Proportion of Tconv that divided at least once at the indicated Treg:Tconv ratios. C and D, Proportion of Tregs that divided at least once at the indicated Treg:Tconv ratios. As expected, Treg divided the most at the lowest Treg:Tnaive ratio where IL-2 is least limiting.
(TIF)

## References

[1] Sun SC (2011) Non-canonical NF-kappaB signaling pathway. Cell Res 21: 71-85. doi:10.1038/cr.2010.177. PubMed: 21173796. 21173796 10.1038/cr.2010.177 PMC3193406

[2] Shinkura R, Kitada K, Matsuda F, Tashiro K, Ikuta K et al. (1999) Alymphoplasia is caused by a point mutation in the mouse gene encoding Nf-kappa b-inducing kinase. Nat Genet 22: 74-77. doi:10.1038/8780. PubMed: 10319865. 10319865 10.1038/8780

[3] Yin L, Wu L, Wesche H, Arthur CD, White JM et al. (2001) Defective lymphotoxin-beta receptor-induced NF-kappaB transcriptional activity in NIK-deficient mice. Science 291: 2162-2165. doi:10.1126/science.1058453. PubMed: 11251123. 11251123 10.1126/science.1058453

[4] Claudio E, Brown K, Park S, Wang H, Siebenlist U (2002) BAFF-induced NEMO-independent processing of NF-kappa B2 in maturing B cells. Nat Immunol 3: 958-965. doi:10.1038/ni842. PubMed: 12352969. 12352969 10.1038/ni842

[5] Boehm T, Scheu S, Pfeffer K, Bleul CC (2003) Thymic medullary epithelial cell differentiation, thymocyte emigration, and the control of autoimmunity require lympho-epithelial cross talk via LTbetaR. J Exp Med 198: 757-769. doi:10.1084/jem.20030794. PubMed: 12953095. 12953095 10.1084/jem.20030794 PMC2194183

[6] Zhu M, Chin RK, Christiansen PA, Lo JC, Liu X et al. (2006) NF-kappaB2 is required for the establishment of central tolerance through an Aire-dependent pathway. J Clin Invest 116: 2964-2971. doi:10.1172/JCI28326. PubMed: 17039258. 17039258 10.1172/JCI28326 PMC1592546

[7] Miyawaki S, Nakamura Y, Suzuka H, Koba M, Yasumizu R et al. (1994) A new mutation, aly, that induces a generalized lack of lymph nodes accompanied by immunodeficiency in mice. Eur J Immunol 24: 429-434. doi:10.1002/eji.1830240224. PubMed: 8299692. 8299692 10.1002/eji.1830240224

[8] Akiyama T, Shimo Y, Yanai H, Qin J, Ohshima D et al. (2008) The tumor necrosis factor family receptors RANK and CD40 cooperatively establish the thymic medullary microenvironment and self-tolerance. Immunity 29: 423-437. doi:10.1016/j.immuni.2008.06.015. PubMed: 18799149. 18799149 10.1016/j.immuni.2008.06.015

[9] Kajiura F, Sun S, Nomura T, Izumi K, Ueno T et al. (2004) NF-kappa B-inducing kinase establishes self-tolerance in a thymic stroma-dependent manner. J Immunol 172: 2067-2075. PubMed: 14764671. 14764671 10.4049/jimmunol.172.4.2067

[10] Aya K, Alhawagri M, Hagen-Stapleton A, Kitaura H, Kanagawa O et al. (2005) NF-(kappa)B-inducing kinase controls lymphocyte and osteoclast activities in inflammatory arthritis. J Clin Invest 115: 1848-1854. doi:10.1172/JCI23763. PubMed: 15937549. 15937549 10.1172/JCI23763 PMC1142111

[11] Jin W, Zhou XF, Yu J, Cheng X, Sun SC (2009) Regulation of Th17 cell differentiation and EAE induction by MAP3K NIK. Blood 113: 6603-6610. doi:10.1182/blood-2008-12-192914. PubMed: 19411637. 19411637 10.1182/blood-2008-12-192914 PMC2710918

[12] Murray SE, Polesso F, Rowe AM, Basak S, Koguchi Y et al. (2011) NF-kappaB-inducing kinase plays an essential T cell-intrinsic role in graft-versus-host disease and lethal autoimmunity in mice. J Clin Invest 121: 4775-4786. doi:10.1172/JCI44943. PubMed: 22045568. 22045568 10.1172/JCI44943 PMC3223068

[13] Xiao X, Balasubramanian S, Liu W, Chu X, Wang H et al. (2012) OX40 signaling favors the induction of T(H)9 cells and airway inflammation. Nat Immunol 13: 981-990. doi:10.1038/ni.2390. PubMed: 22842344. 22842344 10.1038/ni.2390 PMC3806044

[14] Fontenot JD, Gavin MA, Rudensky AY (2003) Foxp3 programs the development and function of CD4(+)CD25(+) regulatory T cells. Nat Immunol 4: 330-336. doi:10.1038/ni904. PubMed: 12612578. 12612578 10.1038/ni904

[15] Khattri R, Cox T, Yasayko SA, Ramsdell F (2003) An essential role for Scurfin in CD4(+)CD25(+) T regulatory cells. Nat Immunol 4: 337-342. doi:10.1038/ni909. PubMed: 12612581. 12612581 10.1038/ni909

[16] Hori S, Nomura T, Sakaguchi S (2003) Control of regulatory T cell development by the transcription factor foxp3. Science 299: 1057-1061. doi:10.1126/science.1079490. PubMed: 12522256. 12522256 10.1126/science.1079490

[17] van der Vliet HJ, Nieuwenhuis EE (2007) IPEX as a result of mutations in FOXP3. Clin Dev Immunol: 89017: 89017 PubMed: 18317533. 10.1155/2007/89017 PMC2248278 18317533

[18] Tamura C, Nakazawa M, Kasahara M, Hotta C, Yoshinari M et al. (2006) Impaired function of dendritic cells in alymphoplasia (aly/aly) mice for expansion of CD25+CD4+ regulatory T cells. Autoimmunity 39: 445-453. doi:10.1080/08916930600833390. PubMed: 17060023. 17060023 10.1080/08916930600833390

[19] Isomura I, Palmer S, Grumont RJ, Bunting K, Hoyne G et al. (2009) c-Rel is required for the development of thymic Foxp3+ CD4 regulatory T cells. J Exp Med 206: 3001-3014. doi:10.1084/jem.20091411. PubMed: 19995950. 19995950 10.1084/jem.20091411 PMC2806473

[20] Ruan Q, Kameswaran V, Tone Y, Li L, Liou HC et al. (2009) Development of Foxp3(+) regulatory T cells is driven by the c-Rel enhanceosome. Immunity 31: 932-940. doi:10.1016/j.immuni.2009.10.006. PubMed: 20064450. 20064450 10.1016/j.immuni.2009.10.006 PMC2807990

[21] Long M, Park SG, Strickland I, Hayden MS, Ghosh S (2009) Nuclear factor-kappaB modulates regulatory T cell development by directly regulating expression of Foxp3 transcription factor. Immunity 31: 921-931. doi:10.1016/j.immuni.2009.09.022. PubMed: 20064449. 20064449 10.1016/j.immuni.2009.09.022

[22] Lu LF, Gondek DC, Scott ZA, Noelle RJ (2005) NF kappa B-inducing kinase deficiency results in the development of a subset of regulatory T cells, which shows a hyperproliferative activity upon glucocorticoid-induced TNF receptor family-related gene stimulation. J Immunol 175: 1651-1657. PubMed: 16034105. 16034105 10.4049/jimmunol.175.3.1651

[23] Rowe AM, Murray SE, Raue H-P, Koguchi Y, Slifka MK et al. (2013) A cell-intrinsic requirement for NF-kappaB-inducing kinase (NIK) in CD4 and CD8 T cell memory. J Immunol. In press 10.4049/jimmunol.1301328 PMC3815446 24006459

[24] Tai X, Cowan M, Feigenbaum L, Singer A (2005) CD28 costimulation of developing thymocytes induces Foxp3 expression and regulatory T cell differentiation independently of interleukin 2. Nat Immunol 6: 152-162. doi:10.1038/ni1160. PubMed: 15640801. 15640801 10.1038/ni1160

[25] Zhang R, Huynh A, Whitcher G, Chang J, Maltzman JS et al. (2013) An obligate cell-intrinsic function for CD28 in Tregs. J Clin Invest 123: 580-593. PubMed: 23281398. 23281398 10.1172/JCI65013 PMC3561819

[26] Sánchez-Valdepeñas C, Martín AG, Ramakrishnan P, Wallach D, Fresno M (2006) NF-kappaB-inducing kinase is involved in the activation of the CD28 responsive element through phosphorylation of c-Rel and regulation of its transactivating activity. J Immunol 176: 4666-4674. PubMed: 16585559. 16585559 10.4049/jimmunol.176.8.4666

[27] Takeda I, Ine S, Killeen N, Ndhlovu LC, Murata K et al. (2004) Distinct roles for the OX40-OX40 ligand interaction in regulatory and nonregulatory T cells. J Immunol 172: 3580-3589. PubMed: 15004159. 15004159 10.4049/jimmunol.172.6.3580

[28] Griseri T, Asquith M, Thompson C, Powrie F (2010) OX40 is required for regulatory T cell-mediated control of colitis. J Exp Med 207: 699-709. doi:10.1084/jem.20091618. PubMed: 20368580. 20368580 10.1084/jem.20091618 PMC2856021

[29] Stephens GL, McHugh RS, Whitters MJ, Young DA, Luxenberg D et al. (2004) Engagement of glucocorticoid-induced TNFR family-related receptor on effector T cells by its ligand mediates resistance to suppression by CD4+CD25+ T cells. J Immunol 173: 5008-5020. PubMed: 15470044. 15470044 10.4049/jimmunol.173.8.5008

[30] van Olffen RW, Koning N, van Gisbergen KP, Wensveen FM, Hoek RM et al. (2009) GITR triggering induces expansion of both effector and regulatory CD4+ T cells in vivo. J Immunol 182: 7490-7500. doi:10.4049/jimmunol.0802751. PubMed: 19494272. 19494272 10.4049/jimmunol.0802751

[31] Hauer J, Püschner S, Ramakrishnan P, Simon U, Bongers M et al. (2005) TNF receptor (TNFR)-associated factor (TRAF) 3 serves as an inhibitor of TRAF2/5-mediated activation of the noncanonical NF-kappaB pathway by TRAF-binding TNFRs. Proc Natl Acad Sci U S A 102: 2874-2879. doi:10.1073/pnas.0500187102. PubMed: 15708970. 15708970 10.1073/pnas.0500187102 PMC549490

[32] Andreakos E, Williams RO, Wales J, Foxwell BM, Feldmann M (2006) Activation of NF-kappaB by the intracellular expression of NF-kappaB-inducing kinase acts as a powerful vaccine adjuvant. Proc Natl Acad Sci U S A 103: 14459-14464. doi:10.1073/pnas.0603493103. PubMed: 16971487. 16971487 10.1073/pnas.0603493103 PMC1599984

[33] MacDonald KP, Kuns RD, Rowe V, Morris ES, Banovic T et al. (2007) Effector and regulatory T-cell function is differentially regulated by RelB within antigen-presenting cells during GVHD. Blood 109: 5049-5057. doi:10.1182/blood-2007-01-067249. PubMed: 17327399. 17327399 10.1182/blood-2007-01-067249

[34] Dougan M, Dougan S, Slisz J, Firestone B, Vanneman M et al. (2010) IAP inhibitors enhance co-stimulation to promote tumor immunity. J Exp Med 207: 2195-2206. doi:10.1084/jem.20101123. PubMed: 20837698. 20837698 10.1084/jem.20101123 PMC2947073

