# Supplementary figures and images for: Cell-Intrinsic Role for NF-kappa B-Inducing Kinase in Peripheral Maintenance but Not Thymic Development of Foxp3+ Regulatory T Cells in Mice
Source: PLoS One. 2013 Sep 20;8(9):e76216. doi: 10.1371/journal.pone.0076216 (PMC3779168; doi:10.1371/journal.pone.0076216)

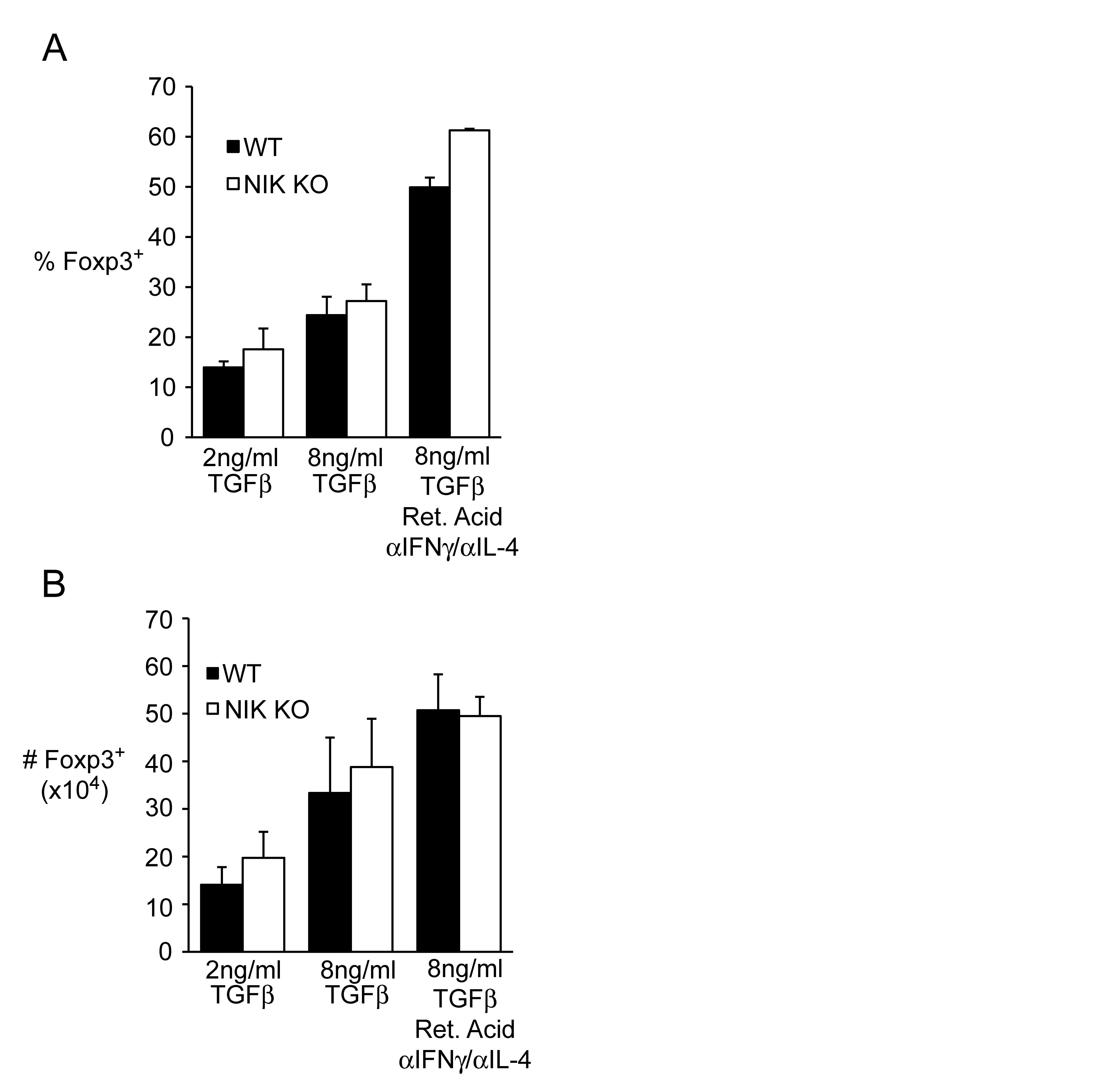

Supplement: Figure S1 — Normal in vitro Treg differentiation from NIK KO conventional T cells. CD25-depleted conventional CD4+ T cells isolated from BM chimeric mice were differentiated in vitro for 3 days under the indicated conditions. In all conditions cells were stimulated with immobilized anti-CD3 + anti-CD28 and supplemented with IL-2. Cultures were analyzed for percent (A) and number (B) of Foxp3 + CD4+ T cells. (TIF) [file pone.0076216.s001.tif]

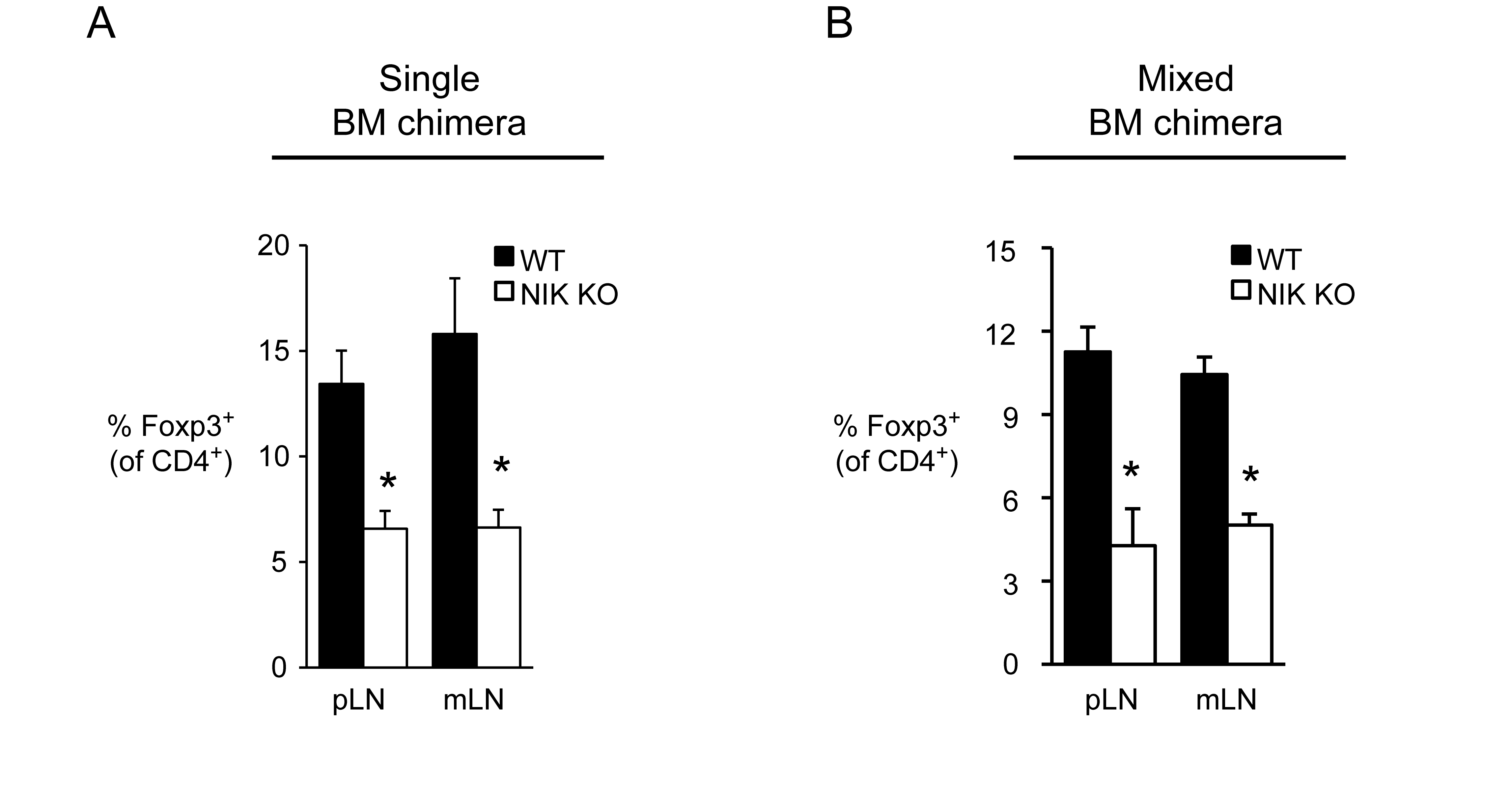

Supplement: Figure S2 — Decreased proportion of NIK KO Tregs in mesenteric and peripheral lymph nodes of single and mixed BM chimeric mice. Single cell suspensions of pooled inguinal, axillary, and brachial LN (pLN) or mesenteric LN (mLN) from single or mixed BM chimeric mice were stained with fluorescently labeled antibodies to CD4, Foxp3, CD45.1, and CD45.2. A, Quantitation of the proportion of CD4+CD45.1- cells that are Foxp3+. B, Quantitation of the proportion of CD4+CD45.1-CD45.2+ cells that are Foxp3+. As in Figure 4, these data compare NIK KO cells with WT littermate control cells. (TIF) [file pone.0076216.s002.tif]

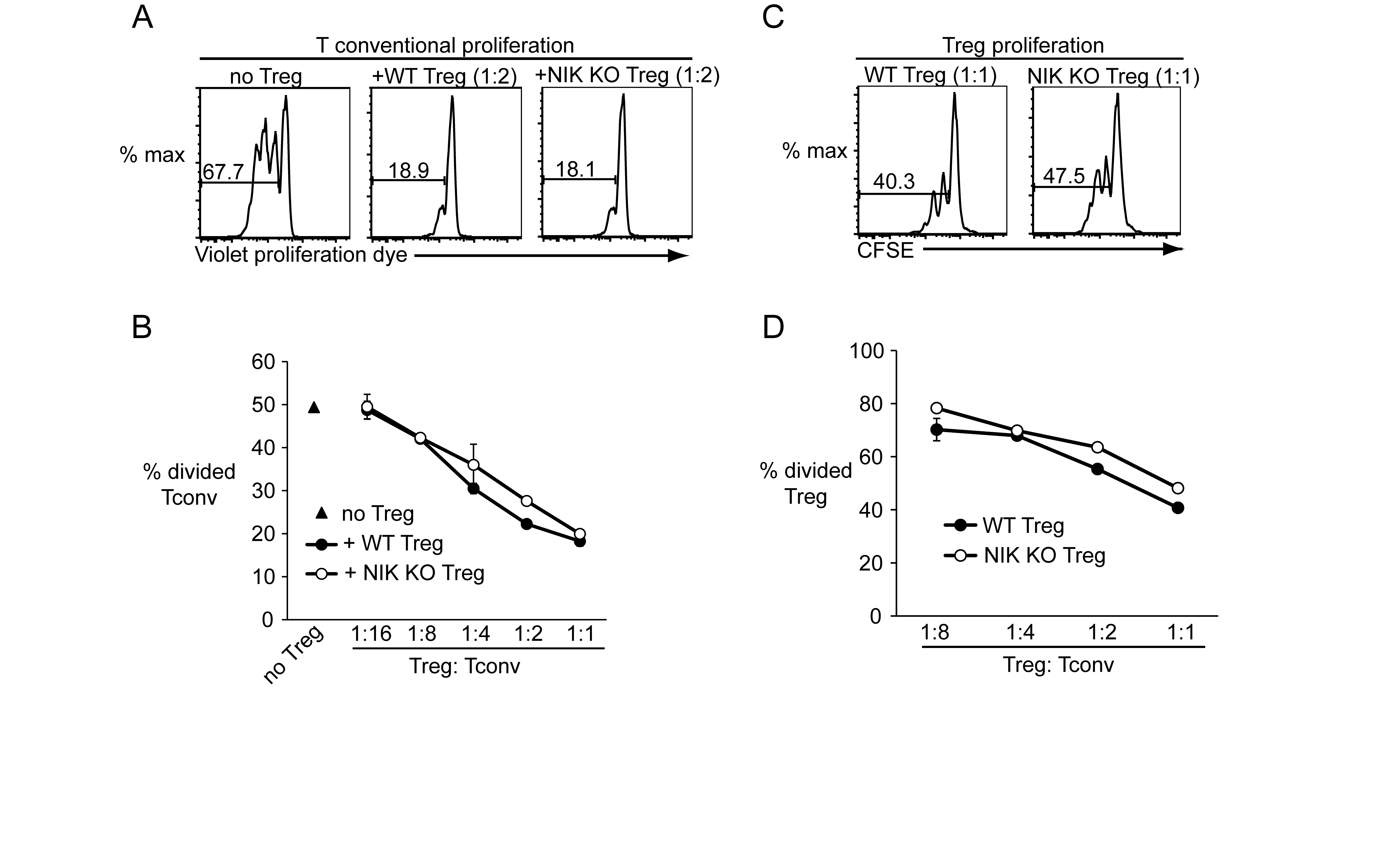

Supplement: Figure S3 — Normal suppressive capacity and proliferation by NIK KO Tregs. Foxp3-RFP + CD4+ cells were FACS-sorted from spleens of WT BM chimera recipients that had received Foxp3-RFP NIK KO or Foxp3-RFP WT BM 8 weeks earlier. Sorted Tregs were labeled with CFSE and plated at varying ratios with CD25-depleted CD4+ Tconv labeled with CellTrace Violet proliferation dye. Cells were stimulated for 3 days with irradiated CD45.1+ splenocytes as APC and soluble anti-CD3. Tconv and Treg cell division was assessed by flow cytometry. A and B, Proportion of Tconv that divided at least once at the indicated Treg:Tconv ratios. C and D, Proportion of Tregs that divided at least once at the indicated Treg:Tconv ratios. As expected, Treg divided the most at the lowest Treg:Tnaive ratio where IL-2 is least limiting. (TIF) [file pone.0076216.s003.tif]
